# Model abstraction for discrete-event systems by binary linear programming with applications to manufacturing systems

**DOI:** 10.1177/00368504211030833

**Published:** 2021-07-22

**Authors:** Lihong Cheng, Lei Feng, Zhiwu Li

**Affiliations:** 1 School of Electro-Mechanical Engineering, Xidian University, Xi’an, China; 2 Department Machine Design, ITM School, KTH Royal Institute of Technology, Stockholm, Sweden

**Keywords:** Discrete-event systems, deterministic finite automata, natural projection, quasi-congruence relation, model abstraction

## Abstract

Model abstraction for finite state automata is helpful for decreasing computational complexity and improving comprehensibility for the verification and control synthesis of discrete-event systems (DES). Supremal quasi-congruence equivalence is an effective method for reducing the state space of DES and its effective algorithms based on graph theory have been developed. In this paper, a new method is proposed to convert the supremal quasi-congruence computation into a binary linear programming problem which can be solved by many powerful integer linear programming and satisfiability (SAT) solvers. Partitioning states to cosets is considered as allocating states to an unknown number of cosets and the requirement of finding the coarsest quasi-congruence is equivalent to using the least number of cosets. The novelty of this paper is to solve the optimal partitioning problem as an optimal state-to-coset allocation problem. The task of finding the coarsest quasi-congruence is equivalent to the objective of finding the least number of cosets. Then the problem can be solved by optimization methods, which are respectively implemented by mixed integer linear programming (MILP) in MATLAB and binary linear programming (BLP) in CPLEX. To reduce the computation time, the translation process is first optimized by introducing fewer decision variables and simplifying constraints in the programming problem. Second, the translation process formulates a few techniques of converting logic constraints on finite automata into binary linear constraints. These techniques will be helpful for other researchers exploiting integer linear programming and SAT solvers for solving partitioning or grouping problems. Third, the computational efficiency and correctness of the proposed method are verified by two different solvers. The proposed model abstraction approach is applied to simplify the large-scale supervisor model of a manufacturing system with five automated guided vehicles. The proposed method is not only a new solution for the coarsest quasi-congruence computation, but also provides us a more intuitive understanding of the quasi-congruence relation in the supervisory control theory. A future research direction is to apply more computationally efficient solvers to compute the optimal state-to-coset allocation problem.

## Introduction

Discrete-event (dynamic) systems are a typical class of computer-integrated man-made system with discrete state spaces and event-driven characteristics. They can be modeled by a state-transition structure such as deterministic or non-deterministic finite automata (DFAs or NFAs) and Petri nets. Both formalisms are used for designing supervisory controllers of a DES.^1–5^ On the one hand, the supervisory control theory developed in Wonham and Cai,^1^ Ramadge and Wonham^3^ is regarded as a seminal work for the treatment of supervisor synthesis for a DES such that the closed-loop behavior is observable,^6,7^ controllable, nonblocking,^8^ and maximally permissive. On the other hand, a Petri net (PN) is also a common mathematical tool for modeling and analyzing a DES, which often avoids explicit state exploration but derives certain network properties through the net structure.^9–14^ Both methods are popular for synthesizing satisfactory supervisors.

Due to the theoretical maturity, DES have received much attention from academy and industry communities.^15–22^ However, DES control methods have not yet been extensively applied to industrial scale problems, owing to the state explosion problem: the number of reachable states increases exponentially with that of concurrent components and variables.^23^ The intractable number of states may quickly deplete the computer memory and terminate the computation if the given system is structurally complex. Therefore, effectively exploring the huge state space with limited memory and time is a significant and persistent research subject.

To tackle the state explosion problem, many reduction methods have been developed, including equivalence reduction^24,25^ and process equivalence,^26^ model abstraction,^27^ symbolic computation and state tree structures,^28,29^ and partial order reduction,^30^ which all benefit from the proper design and analysis of the system architecture.

Model abstraction based on equivalence relations is an efficient way to reduce the state size of finite state automata. An equivalence relation on the state set of a finite state automaton partitions the state set as a collection of equivalence classes (or cosets). The set of cosets defines a simpler finite state automaton with fewer states, which is called the quotient automaton^31,32^ of the original one with respect to the equivalence relation. In many applications, the observable language of the quotient automaton must be equivalent to the original automaton. The identity implies that if two states of the original automaton are equivalent, the same observable event sequences must be defined at both states. This property is guaranteed by the quasi-congruence equivalence,^1^ which is similar to the concept of bi-simulation.^33^

It is necessary to thoroughly investigate the results on the quasi-congruence relation of DES in references,^1,2,34,35^ and use them to effectively reduce the computational complexity of the formal verification and control synthesis of DES. Most contemporary approaches^31,32,36–39^ are based on graph theory. However, we adopt a new approach to the quasi-congruence relations by binary linear programming in the paper, and apply it to reduce the state space.

Wong and Wonham^31^ solve the problem on how to design an observer by modifying the given causal reporter map that is not an observer. The method is to compute the coarsest equivalence relation, which is finer than that of the given map. A polynomial-time algorithm is developed to compute an observer of an automaton, and it can also be used to verify the observer property of natural projection. However, this algorithm cannot be applied to natural projection directly.

Feng and Wonham^32^ adopt the algorithm in Wong and Wonham^31^ to the natural projection, where the minimum event extension is particularly interesting, whose computation, however, is shown to be NP-hard. In this case, an acceptable event extension can be found by developing a polynomial-time algorithm.

These methods are based on graph theory and state-transition data structure. The novelty of this paper is to solve the optimal partitioning problem as an optimal state-to-coset allocation problem. Partitioning states to cosets is considered as allocating states to an unknown number of cosets and the requirement of finding the coarsest quasi-congruence is equivalent to using the least number of cosets. Then the problem can be solved by optimization methods, which leverage many powerful and easy-to-use software tools, such as CPLEX (the CPLEX optimizer can be available on the website https://www.ibm.com/analytics/cplex-optimizer)^40^ and SAT solvers (the SAT solvers can be available on the website https://www.satlive.org/solvers).^41^ These computation tools exploit modern computing technologies, such as graphics processing unit (GPU) computing,^42^ parallel computing,^43^ and cloud computing.^44^ Large scale problems of finding the coarsest quasi-congruence may be solved by modern optimization technologies.

To this end, this paper formalizes the problem of finding the coarsest quasi-congruence as a binary linear programming problem. The design variables form a binary allocation matrix between the states and cosets. The constraints are relationships between states and cosets, and the objective function is to minimize the number of cosets. The main contributions of this paper are summarized below.

1. The number of design variables determines the computational complexity. In Section 4, a property of the allocation problem is discovered and proved to remove around half of the binary design variables.
2. A novel approach on how to compute the supremal quasi-congruence is proposed, which is based on a combination of propositional logic and binary representation in Section 5.
3. The method proposed in Section 5 is converted to a binary linear programming problem, so as to save computing time. In this transformation, we formulate a few techniques of converting logic constraints on finite automata into binary linear constraints which are formalized and proved to be correct in Section 6.
4. The correctness and efficiency of our method are verified by different solvers and examples in Section 8.

Compared with existing studies, the motivation of this work is to propose an optimization method for quasi-congruence computation of DFAs, and to increase the generality of optimal quasi-congruence computation algorithms. The computational results of this method are the same as that of using the computation software TCT (the software TCT can be available on the website https://www.control.utoronto.ca/DES/Research).^45^ for supervisory control design.

The work is structured as follows. The preliminaries pertaining to automata and natural projection are recalled in the following section. Some functions and concepts that are necessary for the quasi-congruence calculation, and the coarsest quasi-congruence are reported in Section 3. Section 7 touches on the main algorithm implementation of the proposed approach. Finally, Section 9 concludes this research.

## Preliminaries

This section recalls some basic concepts of deterministic finite automata used in the paper. Then we review the definition of quasi-congruence relation and some operations on it. More details on automata can be found in references.^1,2,31,32,46–48^

### Fundamental of automata

A *deterministic finite automaton (DFA)*^1^ is represented as a five-tuple,

(1) $$G=(Q,\Sigma ,\delta ,q_{1},Q_{m})$$

-
$Q=\{q_{1},q_{2},\ldots ,q_{n}\}$ is the finite state set and n=|Q| is the number of states in the automaton.
-
Σ is a nonempty finite event set with $\Sigma =\Sigma_{o}\cup \Sigma_{u}$ , where $\Sigma_{o}$ is the observable event set and $\Sigma_{u}$ is the unobservable event set of G .
-
δ:Q×Σ→Q is the state transition function which is considered as a partial function. For an event σ∈Σ , if the function δ(q,σ) is defined, we write it as δ(q,σ)! .
-
$q_{1}\in Q$ is the initial state.
-
$Q_{m}$ is the set of marker states with $Q_{m}\subseteq Q$ .

Note that the set of all finite strings over Σ is denoted as $\Sigma^{*}$ . The empty string ε denotes the string with length 0.

### Natural projection

Given two event sets: Σ is a finite alphabet and $\Sigma_{o}$ is the set of observable events with $\Sigma_{o}\subseteq \Sigma$ , the corresponding *natural projection* function is $P:\Sigma^{*}\to \Sigma_{o}^{*}$ , such that P(ε)=ε and

(2) $$P(\sigma )=\left\{ \begin{array}{c} \varepsilon ,\;\sigma \in \Sigma -\Sigma_{o}\\ \sigma ,\;\sigma \in \Sigma_{o} \end{array}\right.$$

(3) $$P(s\sigma )=P(s)P(\sigma ),\;s\in \Sigma^{*},\sigma \in \Sigma$$

Next, the natural projection P can be extended to the *image function*,^32^ $P:P_{\mathrm{wr}}(\Sigma^{*})\to P_{\mathrm{wr}}(\Sigma_{o}^{*})$ such that

(4) $$\forall L\subseteq \Sigma^{*},\;P(L):=\{P(s)\;|\;s\in L\}.$$

Note that $P_{\mathrm{wr}}(A)$ is the power set of set A . Mathematically, the inverse function of natural projection P does not exist. Here, the notation of the function $P^{-1}$ is expressed as the *inverse image function*, which can be defined as $P^{-1}:P_{\mathrm{wr}}(\Sigma_{o}^{*})\to P_{\mathrm{wr}}(\Sigma^{*})$ such that

(5) $$\forall L_{o}\subseteq \Sigma_{o}^{*},\;P^{-1}(L_{o}):=\{s\in \Sigma^{*}\;|\;P(s)\in L_{o}\}.$$

## Equivalence relation and quasi-congruence relation

This section defines three important functions and some concepts associated with the quasi-congruence computation.

### Equivalence relation

Suppose that E(Q) is the lattice of equivalence relations on the state set Q . Given an *equivalence relation* π∈E(Q) , it must meet the following properties.^1,49^

1.
(∀q∈Q)qπq ( π is reflexive.)
2.
(∀q,q′∈Q)qπq′⇒q′πq ( π is symmetric.)
3.
(∀q,q′,q″∈Q)qπq′∧q′πq″⇒qπq″ ( π is transitive.)

In this paper, qπq′ is also written as q≡q′(modπ) . For any state q∈Q , the *coset* (or *equivalence class*) of q with respect to the equivalence relation π is denoted as $[q{]}_{\pi }$ .

(6) $$[q{]}_{\pi }:=\{q\prime \in Q|\;q\prime \pi q\}\subseteq Q$$

By reflexivity $q\in [q{]}_{\pi }$ , every coset is nonempty. In this paper, Q/π represents the set of all cosets.

### Functions

In a DFA G , we can decide the possibly reachable states from any state $q_{i}\in Q$ based only on observation of the events in $\Sigma_{o}$ .

**Definition 1.** Let $s_{o}\in \Sigma_{o}^{*}$ be an observable string and $q_{i}\in Q$ a state. The function Δ is defined as

(7) $$\Delta :Q\times \Sigma_{o}^{*}\to P_{\mathrm{wr}}(Q)$$

and

$$\Delta (q_{i},s_{o}):=\{q\prime \in Q\;|\;(\exists s\in \Sigma^{*})\;P(s)=s_{o}\wedge q\prime =\delta (q_{i},s)\}.$$

Note that if $\delta (q_{i},s)$ is undefined for every string s with $P(s)=s_{o}$ , then $\Delta (q_{i},s_{o})=Ø$ . In addition, if $s_{o}=\sigma \in \Sigma_{o}$ , we can simplify the notation of $\Delta (q_{i},\sigma )$ to $\Delta_{\sigma }(q_{i})$ , which denotes *the set of reachable states* from state $q_{i}\in Q$ via an observable event σ . Similarly, if $s_{o}=\varepsilon$ , it can be written as $\Delta_{\varepsilon }(q_{i})$ which denotes the set of all reachable states from state $q_{i}\in Q$ via a sequence of unobservable events in $\Sigma -\Sigma_{o}$ . Moreover, *the set of all reachable marker states* can be computed by $\Delta_{m}(q_{i})=\Delta_{\varepsilon }(q_{i})\cap Q_{m}$ .

For concise presentation, we define a new event μ∉Σ , and define $\Delta_{\mu }$ as $\Delta_{m}$ and $\Sigma \prime_{o}=\Sigma_{o}\cup \{\mu \}$ . In addition, we denote n={1,2,⋯,n} .

**Definition 2.** *Let π be an equivalence relation, $q_{i}\in Q$ and $\sigma \in \Sigma_{o}$ . Denote the canonical projection function as $P_{\pi }:Q\to Q/\pi :q_{i}\to [q_{i}{]}_{\pi }$ . We can further define its extension projection function as*

(8) $$P_{\pi }:P_{\mathrm{wr}}(Q)\to P_{\mathrm{wr}}(Q/\pi ).$$

**Definition 3.***Function $P_{\pi }{}^{\circ}\Delta_{\sigma }$ is the composition function of $\Delta_{\sigma }$ and $P_{\pi }$ *,

(9) $$P_{\pi }{}^{\circ}\Delta_{\sigma }:Q\to P_{\mathrm{wr}}(Q/\pi ).$$

At a state $q_{i}\in Q$ , this composed function $P_{\pi }{}^{\circ}\Delta_{\sigma }$ can be further expressed as $P_{\pi }(\Delta_{\sigma }(q_{i})):=\{[q{]}_{\pi }\;|\;q\in \Delta_{\sigma }(q_{i})\}$ .

The composite $\pi {}^{\circ}\Delta_{\sigma }$ ( $\sigma \in \Sigma_{o}^{\prime }$ ) defines an *equivalence relation* on Q as follows. Suppose i≠j and i,j∈n .

$$(\forall q_{i},q_{j}\in Q)\;q_{i}\equiv q_{j}(\mathrm{mod}\;\pi {}^{\circ}\Delta_{\sigma })\Leftrightarrow$$

(10) $$\left\{ \begin{array}{c} (\forall x\in \Delta_{\sigma }(q_{i}))\;(\exists x\prime \in \Delta_{\sigma }(q_{j}))\;x\equiv x\prime (\mathrm{mod}\;\pi );\\ (\forall x\prime \in \Delta_{\sigma }(q_{j}))\;(\exists x\in \Delta_{\sigma }(q_{i}))\;x\prime \equiv x(\mathrm{mod}\;\pi ). \end{array}\right.$$

### Quasi-congruence relation

Assume that (Q,Δ) is a nondeterministic dynamic system where Q is the set of system states and $\Delta :Q\to P_{\mathrm{wr}}(Q)$ is its state transition function. According to references,^1,31,32^ we can define the quasi-congruence relation as follows.

**Definition 4.** *An equivalence relation π on Q is a quasi-congruence for (Q,Δ) , if it satisfies the following three equivalent conditions.*
(1) π≤π°Δ .
(2) $(\forall q_{i},q_{j}\in Q)\;P_{\pi }(q_{i})=P_{\pi }(q_{j})\Rightarrow P_{\pi }{}^{\circ}\Delta (q_{i})=P_{\pi }{}^{\circ}\Delta (q_{j}).$
(3) $(\forall q_{i},q_{j}\in Q)\;q_{i}\equiv q_{j}(\mathrm{mod}\;\pi )\Rightarrow q_{i}\equiv q_{j}(\mathrm{mod}\;\pi {}^{\circ}\Delta ).$

### The coarsest quasi-congruence

**Definition 5.** *Given a DFA G and the observable event set $\Sigma_{o}\subseteq \Sigma$ , the coarsest quasi-congruence* ^31,50,51^ *with respect to Q and $\Sigma_{o}\prime$ is*

(11) $$\rho :=\sup\{\pi \in E(Q)\;|\;\pi \leq (\wedge_{\sigma \in \Sigma_{o}\prime }(\pi {}^{\circ}\Delta_{\sigma }))\}.$$

### Quotient automaton

For a state set Q and the coarsest quasi-congruence relation ρ∈E(Q) , we have the canonical projection $P_{\rho }:Q\to Q/\rho$ as defined in Definition 2. Renaming the cosets in Q/ρ , we get a new state set Q′ and a bijection r:Q/ρ≅Q′ . The composition of $P_{\rho }$ and r is denoted as a function $g=r{}^{\circ}P_{\rho }$ , with equivalence kernel kerg=ρ .

**Definition 6.** *Given a DFA $G=(Q,\Sigma ,\delta ,q_{1},Q_{m})$ , an observable event set $\Sigma_{o}\subseteq \Sigma$ , and an equivalence relation ρ∈E(Q) , the quotient automaton* ^1,51^ *is*

(12) $$G\prime =(Q\;\prime ,\Sigma_{o},\eta ,q\prime_{1},Q\prime_{m})=G/(\Sigma_{o},\rho )$$

where Q′≅Q/ρ , $q\prime_{1}:=g(q_{1})$ , $Q\prime_{m}:=g(Q_{m})$ . Here, the transition function is $\eta :Q\prime \times (\Sigma_{o}\cup \{\varepsilon \})\to P_{\mathrm{wr}}(Q\prime )$ as follows.

$$\eta (q\prime ,\sigma )\mapsto \left\{ \begin{array}{c} g{}^{\circ}\delta (g^{-1}(q\prime ),\sigma ),\;\;\;\;\;\;\;\;\;\;\;\;\;\;\;\;\;\;\;\;\;\;\;\mathrm{if}\;\;\sigma \in \Sigma_{o}\\ g{}^{\circ}\delta (g^{-1}(q\prime ),\Sigma -\Sigma_{o})-\{q\prime \},\;\;\;\;\mathrm{if}\;\;\sigma =\varepsilon \end{array}\right.$$

The quotient automaton may be nondeterministic.

## Matrix representations

Define the *reachable matrix* of each event σ ( $\sigma \in \Sigma_{o}\prime$ ) as a Boolean matrix $R_{\sigma }$ , which is an n×n square matrix and n=|Q| . For any i,j∈n ,

(13) $$R_{\sigma }(i,j)=\left\{ \begin{array}{c} 1,\;q_{j}\in \Delta_{\sigma }(q_{i});\\ 0,\;\mathrm{otherwise}. \end{array}\right.$$

Let π∈E(Q) be a quasi-congruence relation on the state set Q . Assume that π partitions Q into m≤n cosets, then $Q/\pi =\{C_{1},C_{2},\cdots ,C_{m}\}$ and $C_{j}$ represents a coset.

The quasi-congruence relation π is representable by an allocation matrix $X_{n\times m}$ , which is also Boolean, that is, $x_{\mathrm{ij}}=1$ iff $q_{i}\in C_{j}$ , i∈n , j∈m . To decrease the number of unknown variables, we show that through permutation of the cosets, the allocation matrix is reducible to a lower triangular matrix as formalized in Proposition 1.

**Proposition 1.** *There must exist a permutation of the cosets $\left\{C_{1},C_{2},\cdots ,C_{m}\right\}$ such that the allocation matrix $X_{n\times m}$ is lower triangular, namely*

(14) $$(\forall i\in n)\;(\forall j\in m)\;i<j\to x_{\mathrm{ij}}=0.$$

**Proof.** According to the definition of the allocation matrix $X_{n\times m}$ , if m=1 , then there is only one coset $C_{1}$ , so the proposition is true.

If 1<m≤n , then we perform the following operations iteratively for each state $q_{i}\in Q$ in an ascending order of i∈n . If $q_{i}\in C_{j}$ and i≥j , then we need not any permutation of columns, that is, $x_{\mathrm{ij}}=1$ , and the rest of the elements in the i -th row are 0. Otherwise, if $q_{i}\in C_{j}$ and i<j , then $x_{\mathrm{ii}}=0$ and $x_{\mathrm{ij}}=1$ . In this case, since the previous operations have ensured that $(\forall s\in i-1)\;(\forall t\in m)\;s<t\to x_{\mathrm{st}}=0$ , the first i−1 rows of the allocation matrix is already lower triangular.

Now that the i -th row violates the lower triangular property, we permute cosets $C_{i}$ and $C_{j}$ and denote the new allocation matrix as X′ . After the permutation, $x\prime_{\mathrm{ii}}=1$ and $x\prime_{\mathrm{ij}}=0$ . The i -th row satisfies the lower-triangular property. We need to further prove that the first i−1 rows of the new allocation matrix still satisfy the lower triangular property.

For all s∈i−1 and t∈m , if t∉{i,j} , then $x\prime_{\mathrm{st}}=x_{\mathrm{st}}$ . Under the same condition of s , if t=i , then $x\prime_{\mathrm{si}}=x_{\mathrm{sj}}=0$ ; if t=j , then $x\prime_{\mathrm{sj}}=x_{\mathrm{si}}=0$ . These arguments prove that the first i−1 rows of X′ indeed satisfy the lower triangular property.

Set X=X′ and iterate the operations from the next state $q_{i+1}$ . The procedure terminates when the allocation of state $q_{m}$ also satisfies the lower-triangular property.

Finally, we can construct the lower triangle allocation matrix $X_{n\times m}$ as shown in Table 1.

**Table 1.** Lower triangular allocation matrix $X_{n\times m}$ .

$X_{n\times m}$	$C_{1}$	$C_{2}$	…	$C_{m}$
$q_{1}$	$x_{11}$	0	0	0
$q_{2}$	$x_{21}$	$x_{22}$	0	0
⋮	⋮	⋮	⋱	0
$q_{m}$	$x_{m1}$	$x_{m2}$	…	$x_{\mathrm{mm}}$
⋮	⋮	⋮	⋮	⋮
$q_{n}$	$x_{n1}$	$x_{n2}$	…	$x_{\mathrm{nm}}$

## Quasi-congruence calculation

In this part, we propose a new approach of calculating the supremal quasi-congruence using the Boolean allocation matrix and logical propositions.

Let π be an equivalence relation on the state set Q , n=|Q| . Suppose that the number of cosets is m , m≤n . Since the minimal number of cosets is unknown, we define the Boolean variable $y_{j}$ to represent if coset $C_{j}$ is not empty, where j∈m .

(15) $$y_{j}=\left\{ \begin{array}{c} 1,\;\exists \;q\in C_{j};\\ 0,\;\forall \;q\notin C_{j}. \end{array}\right.$$

Then the decision variables are

(16) $$\left\{ \begin{array}{c} x_{\mathrm{ij}}\in \{0,1\},\;1\leq i\leq n,1\leq j\leq m,j\leq i;\\ \;y_{k}\in \{0,1\},\;1\leq k\leq m. \end{array}\right.$$

In total, there are $\frac{m\cdot (2\cdot n-m+1)}{2}+m$ decision variables.

The objective is to find the coarsest quasi-congruence, which has the least number of cosets among all quasi-congruences of the given DFA G based on an observable event set $\Sigma_{o}$ . Therefore the objective function for the optimization problem is:

(17) $$\underset{x_{\mathrm{ij}},y_{k}}{\min}\;\sum_{k=1}^{m}y_{k}.$$

To represent quasi-congruences, these decision variables must satisfy the following constraints from C1 to C3.

**C1:** For each coset $C_{k}$ , $y_{k}=1$ iff at least one state is allocated to it, that is,

(18) $$y_{k}=\vee_{i=k}^{n}\;x_{\mathrm{ik}},\;1\leq k\leq m.$$

**C2:** Each state must belong to one and only one coset, that is,

(19) $$\sum_{j=1}^{\min(i,m)}x_{\mathrm{ij}}=1,\;1\leq i\leq n.$$

**C3:** For all the state pairs $q_{i},q_{j}\in Q$ and all $\sigma \in \Sigma_{o}\prime$ ,

(20) $$q_{i}\equiv q_{j}(\mathrm{mod}\;\pi )\to P_{\pi }{}^{\circ}\Delta_{\sigma }(q_{i})=P_{\pi }{}^{\circ}\Delta_{\sigma }(q_{j}).$$

If two states $q_{i}$ , $q_{j}$ belong to the same coset $C_{k}$ ( 1≤k≤m ), then they must meet the above constraints C3. Equation (20) is further transformed into propositions consisting of binary variables in two steps.

- Step 1: The reachable state sets of states $q_{i}$ and $q_{j}$ can be obtained by standard algorithms, assuming that they are represented as

$$\Delta_{\sigma }(q_{i})=\{q_{i_{1}},q_{i_{2}},\ldots ,q_{i_{p}}\},,\mathrm{where}\;q_{i_{p}}\in Q,1\leq i_{p}\leq n$$

$$\Delta_{\sigma }(q_{j})=\{q_{j_{1}},q_{j_{2}},\ldots ,q_{j_{t}}\},\mathrm{where}\;q_{j_{t}}\in Q,1\leq j_{t}\leq n.$$

Based on the definition of reachable matrix in (13), the two state subsets are equivalent to two row vectors of length n , namely $R_{\sigma }(i,:)$ and $R_{\sigma }(\;j,:)$ , respectively. By (13), $R_{\sigma }(i,k)=1$ if and only if $q_{k}\in \Delta_{\sigma }(q_{i})$ . In the special case $\Delta_{\sigma }(q_{i})=Ø$ , the row vector $R_{\sigma }(i,:)=0_{1\times n}$ holds.

- Step 2: we need to compute $P_{\pi }{}^{\circ}\Delta_{\sigma }(q_{i})$ and $P_{\pi }{}^{\circ}\Delta_{\sigma }(q_{j})$ . The formulas can be simply implemented by Boolean matrix multiplication, denoted by ⋀. The rule is the same as normal matrix multiplication, but replaces multiplication by logic ∧ and addition by logic ∨. Given a state $q_{i}\in Q$ , $\sigma \in \Sigma_{o}\prime$ , $P_{\pi }{}^{\circ}\Delta_{\sigma }(q_{i})$ can be represented as

$$R_{\sigma }(i,:)⋀X_{n\times m}=X(i_{1},:)\vee X(i_{2},:)\vee \ldots \vee X(i_{p},:).$$

Similarly, $P_{\pi }{}^{\circ}\Delta_{\sigma }(q_{j})$ is equivalent to the row vector

$$R_{\sigma }(\;j,:)⋀X_{n\times m}=X(j_{1},:)\vee X(j_{2},:)\vee \ldots \vee X(j_{t},:).$$

Thus, it follows that the equality $P_{\pi }{}^{\circ}\Delta_{\sigma }(q_{i})=P_{\pi }{}^{\circ}\Delta_{\sigma }(q_{j})$ is equivalent to the new vector equality as follows.

(21) $$R_{\sigma }(i,:)⋀X_{n\times m}=R_{\sigma }(j,:)⋀X_{n\times m}.$$

**Remark 1. $R_{\sigma }(i,:)$** *is a 1×n row vector and X is an n×m matrix, then the Boolean multiplication is a 1×m row vector. The notation of $X(i_{p},:)$ means that all the elements of the row correspond to state $q_{i_{p}}$ in the allocation matrix $X_{n\times m}$ .*

Therefore, we can further get the logical formulas of this constraint C3:

- If $R_{\sigma }(i,:)=R_{\sigma }(\;j,:)$ , then the vector equality (21) is always true. So the two states $q_{i}$ and $q_{j}$ must belong to the same coset, that is, X(i,:)=X(j,:) .
- If $R_{\sigma }(i,:)\neq R_{\sigma }(\;j,:)$ , then we further consider two cases.

• One case is that one of the two row vectors $R_{\sigma }(i,:)$ and $R_{\sigma }(\;j,:)$ is zero but not both. Then $q_{i}$ and $q_{j}$ cannot belong to the same coset. Consequently, X(i,:)≠X(j,:) . For all k≤min(i,j,m) ,

(22) $$\neg (x_{\mathrm{ik}}=1\wedge x_{\mathrm{jk}}=1)\Leftrightarrow x_{\mathrm{ik}}=0\vee x_{\mathrm{jk}}=0.$$

• When neither of the two reachable state sets is empty, that is, both $R_{\sigma }(i,:)$ and $R_{\sigma }(\;j,:)$ are not zero, the following condition derived from (20) must be satisfied.

(23) $$\begin{array}{c} {}[X(i,:)=X(\;j,:)]\to \{\wedge_{\sigma \in \Sigma_{o}\prime }[R_{\sigma }(i,:)⋀X_{n\times m}=R_{\sigma }(\;j,:)⋀X_{n\times m}]\}. \end{array}$$

## Transforming logical propositions to linear constraints

Many constraints in Section 5 are logical constraints. Solving this problem is a Constraint Satisfaction Problem (CSP) or SAT problem.^52,53^ A well-known solution to the CSP is to convert the logical constraints into binary linear constraints and to solve the problem by binary linear programming.^54^ There are three main logical propositions that need to be converted equivalently. In this section, we will show how to convert them into equivalent linear inequalities.

**Proposition 2.***If y∈{0,1} and $x_{i}\in \{0,1\}$* ( i∈n ) *are binary variables, then the following two expressions are equivalent.*

(24) $$y=\vee_{i=1}^{n}\;x_{i}\Leftrightarrow \frac{y}{n}\leq \sum_{i=1}^{n}x_{i}\leq n\cdot y$$

**Proof.** (⇒) For the logic proposition, we need to consider two scenarios: If y=0 , then all $x_{i}=0$ , i∈n . Then these values satisfy the linear inequalities, because $\frac{y}{n}=0$ , n·y=0 , and $\sum_{i=1}^{n}x_{i}=0$ .

If y=1 , then there exists at least one $x_{i}=1$ . Therefore, $\frac{y}{n}=\frac{1}{n}$ and n·y=n , and $1\leq \sum_{i=1}^{n}x_{i}\leq n$ . Since $\frac{1}{n}\leq 1$ and y=1 , $\frac{1}{n}\leq 1\leq \sum_{i=1}^{n}x_{i}\leq n\Rightarrow \frac{y}{n}\leq \sum_{i=1}^{n}x_{i}\leq n\cdot y$ .

(⇐) If the linear inequalities on the right-hand side hold, then we need to prove that the left logical proposition is also true.

If y=1 , then $\frac{1}{n}\leq \sum_{i=1}^{n}x_{i}\leq n$ . Since $\frac{1}{n}>0$ , $\sum_{i=1}^{n}x_{i}\geq \frac{1}{n}>0$ . Thus, there exists at least one $x_{i}=1$ in this case. Therefore $\vee_{i=1}^{n}x_{i}=1$ .

On the other hand, if y=0 , then $\sum_{i=1}^{n}x_{i}=0$ . Under this condition, all $x_{i}=0$ , i={1,2,…,n} , thus $\vee_{i=1}^{n}x_{i}=0$ .

Clearly, it can be seen from the above derivation that the logic proposition is also satisfied in the two cases.

Accordingly, we can conclude that this equivalent transformation is always true. □

**Proposition 3.***If $x_{i}\in \{0,1\}$* ( i∈m ), *and $y_{j}\in \{0,1\}$* ( j∈n ) *are binary variables, then the following equivalence holds, where N≥max(m,n) is a positive integer.*

(25) $$\vee_{i=1}^{m}\;x_{i}=\vee_{j=1}^{n}\;y_{j}\Leftrightarrow \frac{1}{N}\cdot \sum_{i=1}^{m}x_{i}\leq \sum_{j=1}^{n}y_{j}\leq N\cdot \sum_{i=1}^{m}x_{i}$$

**Proof.** (⇒) The proof is a simple extension of the proof of Proposition 2. For the left logical formula, we also need to consider two scenarios:

If the logical expressions are 1, at least one $x_{i}$ and at least one $y_{j}$ are 1. Then $1\leq \sum_{i=1}^{m}x_{i}\leq m$ , and $1\leq \sum_{j=1}^{n}y_{j}\leq n\leq N$ . Therefore, $\frac{1}{N}\cdot \sum_{i=1}^{m}x_{i}\leq \frac{m}{N}\leq 1$ and $N\cdot \sum_{i=1}^{m}x_{i}\geq N$ . The two linear inequalities at the right-hand side are both true.

If the logical expressions are 0, then all $x_{i}$ and all $y_{j}$ are 0. Then the linear inequalities are also obviously true.

(⇐) If the right-hand side of Proposition 3 holds, then we need to prove that the left logical formula is also true. There are two cases as below.

1. If all $y_{j}=0$ , then

$$\sum_{j=1}^{n}y_{j}=0\Rightarrow \frac{1}{N}\cdot \sum_{i=1}^{m}x_{i}\leq 0\leq N\cdot \sum_{i=1}^{m}x_{i}$$

Since $\frac{1}{N}>0$ and $x_{i}\in \{0,1\}$ ( i∈m ), we have $\sum_{i=1}^{m}x_{i}=0$ , i.e., all $x_{i}=0$ . Obviously, in this case, the logical formula $\vee_{i=1}^{m}\;x_{i}=\vee_{j=1}^{n}\;y_{j}$ is satisfied.

(2) If there exists at least one $y_{j}=1$ , then

$$1\leq \sum_{j=1}^{n}y_{j}\leq N\cdot \sum_{i=1}^{m}x_{i}\Rightarrow 1\leq N\cdot \sum_{i=1}^{m}x_{i}$$

Since $x_{i}\in \{0,1\}$ ( i∈m ) and N≥max(m,n) is a positive integer. Thus, $\sum_{i=1}^{m}x_{i}\geq \frac{1}{N}>0$ , i.e., there must exist at least one $x_{i}=1$ . Clearly, these values also satisfy the logical formula $\vee_{i=1}^{m}\;x_{i}=\vee_{j=1}^{n}\;y_{j}$ .

Therefore, it can be seen from the above derivation that the Proposition 3 is always true. □

Next, we introduce the following lemma for establishing Proposition 4.

**Lemma 1.** *Let A , B and C be logical propositions. The logical equivalence A→(B∧C)⇔(A→B)∧(A→C) is always valid.*
**Proposition 4.** If $z_{1}$ , $z_{2}$ , $x_{i}\in \{0,1\}$ ( i∈m ), and $y_{j}\in \{0,1\}$ ( j∈n ) are all binary variables, where m , n are both positive integers, then the following logical implication formula is equivalent to the given inequalities, where N≥max(m,n) is a positive integer.

(26) $$\begin{array}{c} \left(z_{1}=1\wedge z_{2}=1)\to (\vee_{i=1}^{m}\;x_{i}=\vee_{j=1}^{n}\;y_{j}\right)\\ \Leftrightarrow \left\{ \begin{array}{c} z_{1}+z_{2}-2\leq \sum_{j=1}^{n}y_{j}-\frac{1}{N}\cdot \sum_{i=1}^{m}x_{i}\\ N\cdot (z_{1}+z_{2}-2)\leq N\cdot \sum_{i=1}^{m}x_{i}-\sum_{j=1}^{n}y_{j} \end{array}\right. \end{array}$$

**Proof.** (⇒) According to Proposition 3, we can first equivalently convert the logical implication into the statement as below.

(27) $$(z_{1}=1\wedge z_{2}=1)\to \frac{1}{N}\cdot \sum_{i=1}^{m}x_{i}\leq \sum_{j=1}^{n}y_{j}\leq N\cdot \sum_{i=1}^{m}x_{i}$$

Second, based on Lemma 1, we can translate the logical implication (27) into the following form.

(28) $$\left\{ \begin{array}{c} (z_{1}=1\wedge z_{2}=1)\to \frac{1}{N}\cdot \sum_{i=1}^{m}x_{i}\leq \sum_{j=1}^{n}y_{j}\\ (z_{1}=1\wedge z_{2}=1)\to \sum_{j=1}^{n}y_{j}\leq N\cdot \sum_{i=1}^{m}x_{i} \end{array}\right.$$

The next step is to show that if the two propositions in (28) hold, then the two inequalities of (26) also hold. The proof has two cases.

1. If both $z_{1}$ and $z_{2}$ are 1, then $z_{1}=1\wedge z_{2}=1$ is true, and (28) yields the inequalities $\frac{1}{N}\cdot \sum_{i=1}^{m}x_{i}\leq \sum_{j=1}^{n}y_{j}\leq N\cdot \sum_{i=1}^{m}x_{i}$ . Since $z_{1}+z_{2}-2=0$ , the two inequalities of (26) obviously hold.
2. Otherwise, then $z_{1}+z_{2}-2\leq -1$ . Since $x_{i}\in \{0,1\}$ , $y_{j}\in \{0,1\}$ and m , n are both positive integers, then we can derive the following inequality set.

(29) $$\left\{ \begin{array}{c} 0\leq \sum_{i=1}^{m}x_{i}\leq m\\ 0\leq \sum_{j=1}^{n}y_{j}\leq n \end{array}\right.$$

Because N is a positive integer, $-\frac{1}{N}<0$ . Further, we can transform the above inequality set (29) into the following two inequality pairs.

(30) $$\left\{ \begin{array}{c} -\frac{m}{N}\leq -\frac{1}{N}\cdot \sum_{i=1}^{m}x_{i}\leq 0\\ 0\leq \sum_{j=1}^{n}y_{j}\leq N \end{array}\right.$$

(31) $$\left\{ \begin{array}{c} 0\leq N\cdot \sum_{i=1}^{m}x_{i}\leq N\cdot m\\ -n\leq -\sum_{j=1}^{n}y_{j}\leq 0 \end{array}\right.$$

Summing the inequality pairs in (30) and (31), we have the following two inequalities.

(32) $$\left\{ \begin{array}{c} -\frac{m}{N}\leq (\sum_{j=1}^{n}y_{j}-\frac{1}{N}\cdot \sum_{i=1}^{m}x_{i})\leq N\\ -n\leq (N\cdot \sum_{i=1}^{m}x_{i}-\sum_{j=1}^{n}y_{j})\leq N\cdot m \end{array}\right.$$

Since m,n>0 and N≥max(m,n) , we have $-1\leq -\frac{m}{N}$ and −N≤−n . From this we derive the following pairs.

(33) $$\left\{ \begin{array}{c} -1\leq (\sum_{j=1}^{n}y_{j}-\frac{1}{N}\cdot \sum_{i=1}^{m}x_{i})\leq N\\ -1\cdot N\leq (N\cdot \sum_{i=1}^{m}x_{i}-\sum_{j=1}^{n}y_{j})\leq N\cdot m \end{array}\right.$$

Recalling $z_{1}+z_{2}-2\leq -1$ , then both inequalities in (26) are also satisfied, regardless of the values of $x_{i}$ and $y_{j}$ .

(⇐) We prove this direction in two cases.

1. If $z_{1}=0$ or $z_{2}=0$ , the proposition $z_{1}=1\wedge z_{2}=1$ is always false. The logical proposition at the left-hand side is always true regardless of the values of $x_{i}$ and $y_{j}$ in this case.
2. If $z_{1}=1\wedge z_{2}=1$ , then $z_{1}+z_{2}-2=0$ . In this case, we can convert the set of inequalities in Proposition 4 into the set of inequalities below.

(34) $$\left\{ \begin{array}{c} 0\leq \sum_{j=1}^{n}y_{j}-\frac{1}{N}\cdot \sum_{i=1}^{m}x_{i}\\ 0\leq N\cdot \sum_{i=1}^{m}x_{i}-\sum_{j=1}^{n}y_{j} \end{array}\right.$$

(35) $$\Leftrightarrow \frac{1}{N}\cdot \sum_{i=1}^{m}x_{i}\leq \sum_{j=1}^{n}y_{j}\leq N\cdot \sum_{i=1}^{m}x_{i}$$

The inequalities (35) are equivalent to the right-hand side inequalities by Proposition 3.

Therefore, we can know that the equivalent conversion of Proposition 4 is always true. □

## The main algorithm and procedure of model abstraction

### Main algorithm

According to Proposition 2, it is easy to convert the constraints of C1 into the following pairs of linear inequalities. For any k∈m ,

(36) $$\left\{ \begin{array}{c} \frac{1}{n}\cdot y_{k}-\sum_{i=k}^{n}x_{\mathrm{ik}}\leq 0\\ \sum_{i=k}^{n}x_{\mathrm{ik}}-n\cdot y_{k}\leq 0 \end{array}\right.$$

Moreover, the constraints of C2 are already linear equality constraints. Finally, all linear inequality constraints of C3 are obtained by Algorithm 1.

**Table:** 

Algorithm 1:Computation of the constraints C3
Input: n , m , $(R_{\sigma }{)}_{n\times n}$ for each event $\sigma \in \Sigma \prime_{o}$ , and $X_{n\times m}$ ( m≤n ).
Output: List of linear inequalities corresponding to the constraints C3.
1 **for i=1ton−1 do**
2 **for j=i+1ton do**
3 **for***all* $\sigma \in \Sigma \prime_{o}$
4 **if $R_{\sigma }(i,:)=0_{1\times n}\wedge R_{\sigma }(j,:)\neq 0_{1\times n}$ ** or $R_{\sigma }(i,:)\neq 0_{1\times n}\wedge R_{\sigma }(j,:)=0_{1\times n}$ **then**
5 **for k=1tomin(i,m) do**
6 $x_{\mathrm{ik}}+x_{\mathrm{jk}}\leq 1$
7 **end**
8 goto line 25; /*Iterate to the next j */
9 **end**
10 **end**
11 **for***all* $\sigma \in {\Sigma^{\prime }}_{o}$ **do**
12 **if** $R_{\sigma }(i,:)==R_{\sigma }(j,:)$ **then**
13 continue; /*Iterate to the next event*/
14 **else**
15 $\mathrm{co}l_{i}=\{s\in n\;|\;R_{\sigma }(i,s)=1\}$
16 $\mathrm{co}l_{j}=\{s\prime \in n\;|\;R_{\sigma }(j,s\prime )=1\}$
17 **for k=1tomin(i,m) do**
18 **for t=1tom do**
19 $x_{\mathrm{ik}}+x_{\mathrm{jk}}+\frac{1}{n}\cdot \sum_{s\in \mathrm{co}l_{i}}x_{\mathrm{st}}-\sum_{s\prime \in \mathrm{co}l_{j}}x_{s\prime t}\leq 2$
20 $n\cdot x_{\mathrm{ik}}+n\cdot x_{\mathrm{jk}}-n\cdot \sum_{s\in \mathrm{co}l_{i}}x_{\mathrm{st}}+\sum_{s\prime \in \mathrm{co}l_{j}}x_{s\prime t}\leq 2\cdot n$
21 **end**
22 **end**
23 **end**
24 **end**
25 **end**
26 **end**

In the following Algorithm 1, n is the number of states, m is the maximal number of cosets, $(R_{\sigma }{)}_{n\times n}$ represents the reachable matrices for each event $\sigma \in \Sigma \prime_{o}$ , and $X_{n\times m}$ ( m≤n ) is the allocation matrix we supposed.

This algorithm computes the linear inequalities for all pairs of states $q_{i}$ and $q_{j}$ according to (20). Line 4 determines whether the two states cannot be equivalent. In that case, the algorithm adds the inequalities specified by lines 5–7, skips the remaining part, and iterates to the next value of j . Otherwise, the algorithm continues into the loop of lines 11–24. Lines 12–13 check whether the reachable state sets from both states $q_{i}$ and $q_{j}$ are equivalent. If they are, no constraint is added; otherwise, the algorithm continues to execute lines 15–22 and output the linear inequalities of lines 17–22.

In the algorithm, line 6 corresponds to formula (22) in Section 5. Line 12 represents case (a) of the constraints C3. Lines 14–23 implement the second case in case (b) of C3. Finally, lines 17–22 correspond to the linear inequalities from the transformation of the logical formula (23) according to Proposition 4.

### Model abstraction

This section presents the complete procedure of computing the model abstraction.

Input: A finite automaton $G=(Q,\Sigma ,\delta ,q_{1},Q_{m})$ and an observable event set $\Sigma_{o}\subseteq \Sigma$ .

Step 1: Compute the reachable state set function $\Delta_{\sigma }$ for all $\sigma \in \Sigma_{o}\prime$ . According to the definition of reachable matrix in (13), the reachable matrix of each event σ can be obtained as $(R_{\sigma }{)}_{n\times n}$ .
Step 2: Compute the coarsest quasi-congruence by solving the optimization problem (17)–(20) using the proposed method in the paper, based on all the reachable matrices $(R_{\sigma }{)}_{n\times n}$ and $\sigma \in \Sigma_{o}\prime$ .
Step 3: Compute the quotient automaton for G with the coarsest quasi-congruence, according to Definition 6.

Output: The quotient automaton is the abstraction of G .

## Computational examples

### An illustrative example

**Figure 1.**
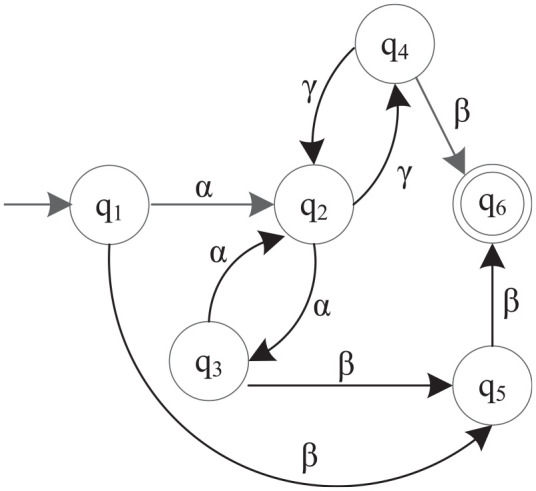
Automaton M2, and an observable event set $\Sigma_{o}=\{\alpha ,\gamma \}$ .

This section elaborates the complete calculation process of the method with the simple example in Figure 1. The automaton M2 has the state set $Q=\{q_{1},q_{2},\ldots ,q_{6}\}$ , the event set Σ={α,β,γ} , the initial state $q_{1}$ and the marker state set $\left\{q_{6}\right\}$ . Given an observable event set $\Sigma_{o}=\{\alpha ,\gamma \}$ , we calculate the coarsest quasi-congruence through the following steps. First, we need to calculate its reachable matrices for all observable events, including $(R_{\alpha }{)}_{6\times 6}$ , $(R_{\gamma }{)}_{6\times 6}$ , and $(R_{\mu }{)}_{6\times 6}$ as in Tables 2 to 4. Second, let m=6 and the corresponding lower triangular allocation matrix $X_{6\times 6}$ is in Table 5. Third, on the basis of Propositions 2–4 and the constraints C1–C3 in Section 5, we can list all linear inequalities constraints for M2 as follows. Here, n=6 and m=6 .

C1: $y_{k}=\vee_{i=k}^{n}\;x_{\mathrm{ik}},\;(k\in m)\Leftrightarrow$

$$\left\{ \begin{array}{c} \frac{1}{n}\cdot y_{k}-x_{\mathrm{kk}}-x_{(k+1)k}-\cdots -x_{\mathrm{nk}}\leq 0\\ x_{\mathrm{kk}}+x_{(k+1)k}+\cdots +x_{\mathrm{nk}}-n\cdot y_{k}\leq 0 \end{array}\right.$$

C2: $\sum_{j=1}^{\min(i,m)}x_{\mathrm{ij}}=1,\;(i\in n)\Leftrightarrow$

$$\left\{ \begin{array}{c} x_{i1}+x_{i2}+\cdots +x_{\mathrm{ii}}=1,\;i<m\\ x_{i1}+x_{i2}+\cdots +x_{\mathrm{im}}=1,\;i\geq m \end{array}\right.$$

C3: For all pairs of i∈n−1 and i+1≤j≤n , for all $\sigma \in \Sigma \prime_{o}$ , we need to consider the following three cases in turn. Firstly, if one of the two row vectors $R_{\sigma }(i,:)$ and $R_{\sigma }(\;j,:)$ is zero but not both, then states $q_{i}$ and $q_{j}$ cannot belong to the same coset. Then we add the following inequalities.

(37) $$x_{\mathrm{ik}}+x_{\mathrm{jk}}\leq 1,\;1\leq k\leq \min(i,m)$$

**Table 2.** Reachable matrix $(R_{\alpha }{)}_{6\times 6}$ of M2.

$(R_{\alpha }{)}_{6\times 6}$	$q_{1}$	$q_{2}$	$q_{3}$	$q_{4}$	$q_{5}$	$q_{6}$
$q_{1}$	0	1	0	0	0	0
$q_{2}$	0	0	1	0	1	1
$q_{3}$	0	1	0	0	0	0
$q_{4}$	0	0	0	0	0	0
$q_{5}$	0	0	0	0	0	0
$q_{6}$	0	0	0	0	0	0

**Table 3.** Reachable matrix $(R_{\gamma }{)}_{6\times 6}$ of M2.

$(R_{\gamma }{)}_{6\times 6}$	$q_{1}$	$q_{2}$	$q_{3}$	$q_{4}$	$q_{5}$	$q_{6}$
$q_{1}$	0	0	0	0	0	0
$q_{2}$	0	0	0	1	0	1
$q_{3}$	0	0	0	0	0	0
$q_{4}$	0	1	0	0	0	0
$q_{5}$	0	0	0	0	0	0
$q_{6}$	0	0	0	0	0	0

**Table 4.** Reachable matrix $(R_{\mu }{)}_{6\times 6}$ of M2.

$(R_{\mu }{)}_{6\times 6}$	$q_{1}$	$q_{2}$	$q_{3}$	$q_{4}$	$q_{5}$	$q_{6}$
$q_{1}$	0	0	0	0	0	1
$q_{2}$	0	0	0	0	0	0
$q_{3}$	0	0	0	0	0	1
$q_{4}$	0	0	0	0	0	1
$q_{5}$	0	0	0	0	0	1
$q_{6}$	0	0	0	0	0	1

**Table 5.** Supposed matrix $X_{6\times 6}$ of M2.

$X_{6\times 6}$	$C_{1}$	$C_{2}$	$C_{3}$	$C_{4}$	$C_{5}$	$C_{6}$
$q_{1}$	$x_{11}$	0	0	0	0	0
$q_{2}$	$x_{21}$	$x_{22}$	0	0	0	0
$q_{3}$	$x_{31}$	$x_{32}$	$x_{33}$	0	0	0
$q_{4}$	$x_{41}$	$x_{42}$	$x_{43}$	$x_{44}$	0	0
$q_{5}$	$x_{51}$	$x_{52}$	$x_{53}$	$x_{54}$	$x_{55}$	0
$q_{6}$	$x_{61}$	$x_{62}$	$x_{63}$	$x_{64}$	$x_{65}$	$x_{66}$

For example, we find $R_{\alpha }(q_{2},:)\neq 0_{1\times n}$ and $R_{\alpha }(q_{4},:)=0_{1\times n}$ in Table 2. According to (37), we have i=2 , j=4 , and k∈{1,2} . The following two inequalities must be added.

$$\left\{ \begin{array}{c} x_{21}+x_{41}\leq 1\\ x_{22}+x_{42}\leq 1 \end{array}\right.$$

Secondly, if the condition $R_{\sigma }(i,:)=R_{\sigma }(\;j,:)$ is true for all reachable matrices ( $\sigma \in \Sigma \prime_{o}$ ), then the two states $q_{i}$ and $q_{j}$ must belong to a coset, and no constraint needs to be added. From Tables 2 to 4, we can see that $\left[R_{\alpha }(1,:)=R_{\alpha }(3,:)]\wedge [R_{\gamma }(1,:)=R_{\gamma }(3,:)]\wedge [R_{\mu }(1,:)=R_{\mu }(3,:)\right]$ , so we can determine that $q_{1}$ and $q_{3}$ must belong to a coset, and there are no constraints to be added in this case. The final case is that $R_{\sigma }(i,:)\neq R_{\sigma }(\;j,:)$ and both are not zero vectors. Since M2 is simple, the third case does not exist in this example. If there is such a case in more complex automata, we also need to add the following pairs of linear inequalities according to (23).

(38) $$\left\{ \begin{array}{c} x_{\mathrm{ik}}+x_{\mathrm{jk}}+\frac{1}{n}\sum_{t=1}^{m}Z_{\sigma }(i,t)-\sum_{t=1}^{m}Z_{\sigma }(\;j,t)\leq 2\\ n(x_{\mathrm{ik}}+x_{\mathrm{jk}})-n\sum_{t=1}^{m}Z_{\sigma }(i,t)+\sum_{t=1}^{m}Z_{\sigma }(\;j,t)\leq 2n \end{array}\right.$$

Note that the symbol $Z_{\sigma }(i,t)$ in the formula (38) represents the new vector multiplication, which can be calculated according to formula (21) and expressed as follows.

(39) $$Z_{\sigma }(i,t)=R_{\sigma }(i,:)⋀X\;(:,t)$$

(40) $$=\vee_{s=1}^{n}[R_{\sigma }(i,s)\wedge X(s,t)]$$

Here, $\sigma \in \Sigma \prime_{o}$ , i∈n and t∈m . On this basis, we can compute the corresponding product matrix $(Z_{\sigma }{)}_{n\times m}$ . For example,

$$Z_{\alpha }(2,3)=\vee_{s=1}^{6}[R_{\sigma }(2,s)\wedge X(s,3)]=x_{33}\vee x_{53}\vee x_{63}.$$

Thus, if k∈m , then

$$Z_{\alpha }(2,:)=R_{\alpha }(2,:)⋀X(:,k)={\left[\begin{array}{c} x_{31}\vee x_{51}\vee x_{61}\\ x_{32}\vee x_{52}\vee x_{62}\\ x_{33}\vee x_{53}\vee x_{63}\\ x_{54}\vee x_{64}\\ x_{55}\vee x_{65}\\ x_{66} \end{array}\right]}_{6\times 1.}^{T}$$

Therefore, this example is formalized as the following binary linear programming problem. The objective function for the optimization problem is:

(41) $$\underset{x_{\mathrm{ij}},y_{k}}{\min}\;\sum_{k=1}^{m}y_{k},\;n=m=6$$

subject to the following constraints.

(42) $$\frac{1}{n}\cdot y_{k}-x_{\mathrm{kk}}-x_{(k+1)k}-\cdots -x_{\mathrm{nk}}\leq 0,\;k\in m$$

(43) $$x_{\mathrm{kk}}+x_{(k+1)k}+\cdots +x_{\mathrm{nk}}-n\cdot y_{k}\leq 0,\;k\in m$$

(44) $$x_{i1}+x_{i2}+\cdots +x_{\mathrm{ii}}=1,\;i\in n$$

(45) $$x_{2k}+x_{\mathrm{jk}}\leq 1,\;j\in \{1,3,4,5,6\},1\leq k\leq \min(2,j)$$

(46) $$x_{4k}+x_{\mathrm{jk}}\leq 1,\;j\in \{1,2,3,5,6\},1\leq k\leq \min(4,j)$$

(47) $$x_{\mathrm{ij}}\in \{0,1\},\;i\leq j\in n$$

(48) $$y_{k}\in \{0,1\},\;k\in m$$

**Figure 2.**
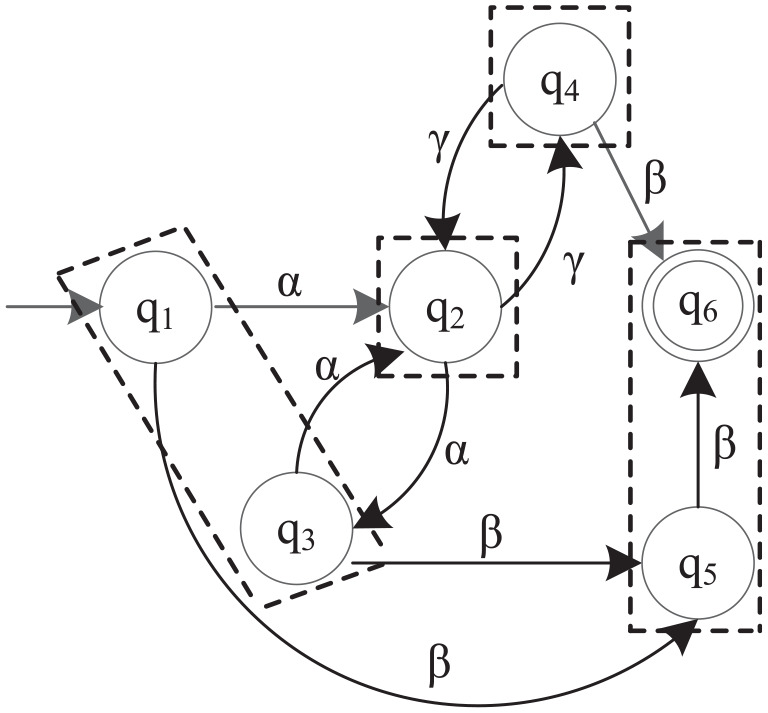
M2 model abstraction based on $\Sigma_{o}=\{\alpha ,\gamma \}$ .

**Figure 3.**
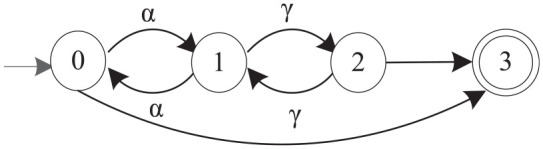
The quotient automaton of M2 with respect to $\Sigma_{o}=\{\alpha ,\gamma \}$ .

In total, this simple example has 27 variables, 6 linear equalities and 35 linear inequalities. The number of linear inequalities obtained by C3 is 23. The problem is solved by MATLAB with a PC with Intel(R) i7-4600U CPU @2.10 GHz, 2.70 GHz, and 16.0 GB installed memory (RAM). The computation time is 0.0135 s. Table 6 shows the final result of the coarsest quasi-congruence relation for M2, and its state partition is $\pi =\{\{q_{1},q_{3}\},\{q_{2}\},\{q_{4}\},\{q_{5},q_{6}\}\}$ , as shown in Figure 2. All the states in a dotted box represent a coset. Figure 3 further illustrates the simplified quotient automaton of M2. The abstracted model is a nondeterministic finite automaton, because there are unobservable transitions from states 0 and 2 to state 3. This suggests that the selected observable event set is not proper for simplifying the original DFA as a smaller DFA. In this case, we may need to consider re-selecting an observable event set.^32^ Finally, the coarsest quasi-congruence is also computed by the standard method in TCT and the result confirms the correctness of the new BLP approach.

**Table 6.** Allocation matrix $X_{6\times 6}$ of M2.

$X_{6\times 6}$	$C_{1}$	$C_{2}$	$C_{3}$	$C_{4}$	$C_{5}$	$C_{6}$
$q_{1}$	1	0	0	0	0	0
$q_{2}$	0	1	0	0	0	0
$q_{3}$	1	0	0	0	0	0
$q_{4}$	0	0	1	0	0	0
$q_{5}$	0	0	0	1	0	0
$q_{6}$	0	0	0	1	0	0

### Comparisons of computation methods

This section compares the computation results of three different methods: the classical graph-partition method^1,45^ implemented in TCT, and the binary linear programming method proposed in this paper which are respectively implemented by mixed integer linear programming (MILP)^55^ in MATLAB and binary linear programming in CPLEX (BLP).

Table 7 is a comparison of computing the supremal quasi-congruence relations on the basis of different observable event sets of the automaton M2 by using the stated three methods. Column 1 lists different sets of observable events. Column 2 lists the result using the graph-based partition method implemented in TCT^45^ for each $\Sigma_{o}$ . Column 3 lists the assumed m value, where each m is equal to 6 for comparison of calculation results. Column 4 shows the size of the linear inequalities C3 obtained by Algorithm 1. Since the size of constraints C3 (Number of linear inequalities × Number of variables) is the main factor that affects the computation time of the method proposed in the paper, it is necessary to analyze the relationship between its size and running time. Columns 5–6 show the running time using two different methods, the MILP method in MATLAB and the BLP method implemented by calling CPLEX Studio 12.8 in MATLAB. Note that when the running time of BLP method is less than 0.01 s, the system automatically reduces it to 0.00 s. The computation results are summarized as follows.

- The computational results using MILP and BLP methods are the same as that of using the computation software TCT.
- On different sets of observable events, when m is invariant, the scale of linear inequalities C3 is not necessarily the same.
- In general, however, the smaller the constraint size is, the shorter the run time will be. Moreover, the smaller the number of observable events is, the smaller the number of cosets will be.

**Table 7.** Computation results of M2 based on different $\Sigma_{o}$ .

$\Sigma_{o}$	TCT	m	Size of C3	Time/s	
				MILP	BLP
{α,β,γ}	5	6	34×27	0.0171	0.01
{α,β}	4	6	193×27	0.0237	0.01
{β,γ}	4	6	169×27	0.0304	0.01
{α,γ}	4	6	29×27	0.0135	0.01
{α}	2	6	104×27	0.0142	0.00
{β}	3	6	135×27	0.0159	0.00
{γ}	2	6	92×27	0.0151	0.00

Table 8 summarizes the computation results for a few large examples. Column 1 lists the automata of different sizes, including the corresponding size of state space n , the size of event set |Σ| and different observable event sets $\Sigma_{o}$ . Columns 2–3 respectively list the real number of cosets of the coarsest quasi-congruence |QC| and the number of cosets assumed m ( m≤n ). Column 4 shows the size of constraints C3 (Number of linear inequalities × Number of variables). Columns 5–6 show the calculation time for two different methods: MILP method and BLP method. Each line represents the results of different m assumed for the same automaton. In the first column of Table 8, M3 and M4 are created by ourselves to test the algorithm. M3 has 21 states, 22 events, and 26 transitions. M4 has 54 states, 53 events, and 100 transitions. We have the following observations from Table 8.

- For the same automaton and the same observable event set, a tight estimate of m is important. A smaller m generally results in less computation time. On the other hand, if m is less than the real minimum, the binary linear programming problem is infeasible.
- As the state space increases, the calculation time and memory consumption of MILP method also increase, mainly due to the increase of the number of linear inequality constraints C3. When the automaton is not large, the MILP method is efficient.
- The BLP method is more efficient than MILP method under the same conditions.

**Table 8.** Comparison of calculation results.

Example	|QC|	m	Size of C3	Time/s	
				MILP	BLP
SMALLFACT^32^		12	157×90	0.0503	0.02
n=12 , |Σ|=9	3	6	129×63	0.0302	0.02
$\Sigma_{o}=\{12,22,32\}$		3	78×36	0.0204	0.00
M3		21	1261×252	73.0757	0.06
n=21 , |Σ|=22	8	10	1092×175	8.5508	0.05
$\Sigma_{o}=\{13,31,53,71,81\}$		8	975×148	1.6661	0.03
M3		21	3312×252	55.7098	1.66
n=21 , |Σ|=22	8	10	1620×175	10.6396	0.42
$\Sigma_{o}=\{15,33,55,75\}$		8	1310×148	2.4277	0.25
M3		21	995×252	0.1757	0.02
n=21 , |Σ|=22	3	10	905×175	0.1579	0.01
$\Sigma_{o}=\{51\}$		3	387×63	0.0249	0.01
M4		54	8121×1539	1.7758	0.19
n=54 , |Σ|=53	3	27	6750×1134	1.4337	0.17
$\Sigma_{o}=\{15,25,35,45\}$		3	1002×162	0.0248	0.02
M4		54	10446×1539	35.7073	0.38
n=54 , |Σ|=53	4	10	3885×505	0.9188	0.11
$\Sigma_{o}=\{28,38,48\}$		4	1662×214	0.1867	0.05
M4		54	19519×1539	7209.08	1.05
n=54 , |Σ|=53	10	27	16969×1134	7200.51	0.90
$\Sigma_{o}=\{10,16,41,50,52\}$		10	9350×505	7199.90	0.66
FMS^50^					
n=180 , |Σ|=13					
$\Sigma_{o}=\{211,231,241\}$	3	3	276732×540	328.5743	102.05
$\Sigma_{o}=\{230,251,371\}$	6	6	877048×1071	10,138.733	901.29
PRODCELL^28^					
n=2478 , |Σ|=23	2	2	1503438×4957	18.3476	0.94
$\Sigma_{o}=\{610\}$					

### An application

**Figure 4.**
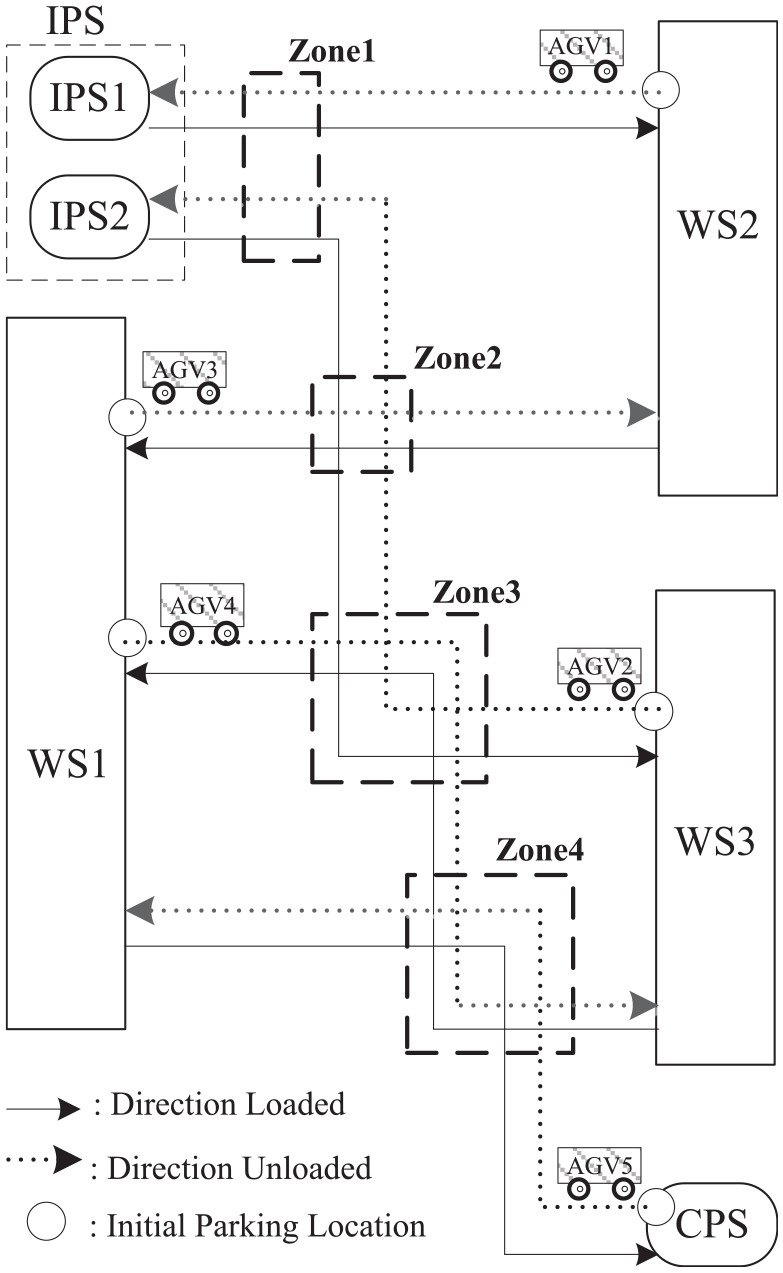
The AGVs system.

The proposed model abstraction method is applied to simplify the supremal nonblocking supervisor of a manufacturing system with five automated guided vehicles (AGVs),^1,51^ as shown in Figure 4. The simplified supervisor model exposes the control logic hidden in the original large automaton model. The manufacturing system has two input stations IPS1 and IPS2 for parts of types 1, 2; three workstations WS1, WS2, and WS3, each containing a conveyor belt; one completed parts station CPS; and five AGVs. The five AGVs alternately load and unload parts on their own fixed circular routes.

The system model consists of five automaton models of the five AGVs, eight automaton models of control specifications.

**Figure 5.**
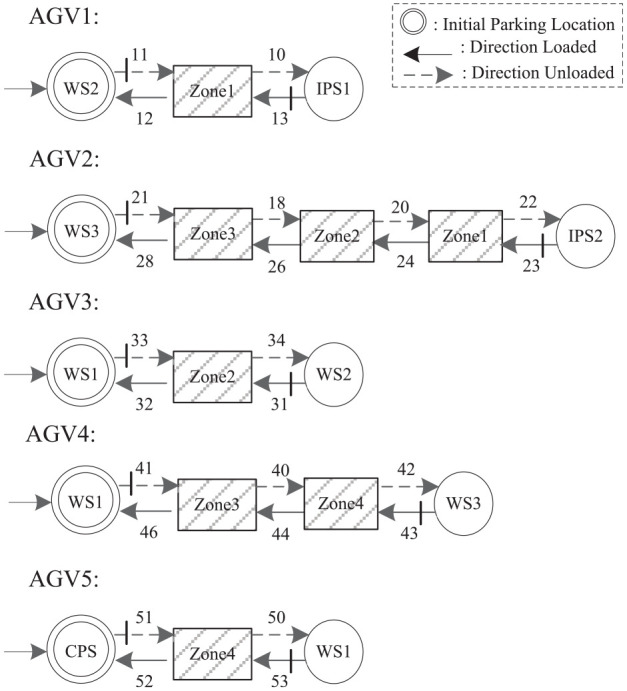
The automaton models for the five AGVs.

- Figure 5 shows the automaton models of the five AGVs. The states corresponding to the physical zones are illustrated as gray rectangles. The solid and dotted lines represent the loading and unloading of each AGV respectively.
- Each of the four shared zones is modeled as an automaton ( $\mathrm{Zon}e_{j}$ , j=1,⋯,4 ) that restricts only one AGV in the zone.
- Each of the three workstations is modeled as an automaton ( $WS_{k}$ , k=1,⋯,3 ). WS1 receives type 1 parts via AGV3 and type 2 parts via AGV4, and assembles both into the complete products, which are transferred to CPS via AGV5. WS2 receives type 1 parts via AGV1 and processes them. The finished type 1 parts are transferred to WS1 via AGV3. WS3 receives type 2 parts via AGV2 and processes them. The finished type 2 parts are transferred to WS1 via AGV4.
- The dotted zone of IPS is modeled as an automaton, which restricts only one AGV inside the zone.

More details of the AGVs system can be found in Wonham and Cai,^1^ Feng and Wonham.^51^ The plant model is the synchronous product of the five AGV models. Note that the two values in parentheses respectively represent the number of states and transitions in the automaton model obtained by each calculation.

$$\mathrm{AGV}:=\mathrm{AG}V_{1}||\mathrm{AG}V_{2}||\cdots ||\mathrm{AG}V_{5}\;(3072,15360).$$

**Figure 6.**
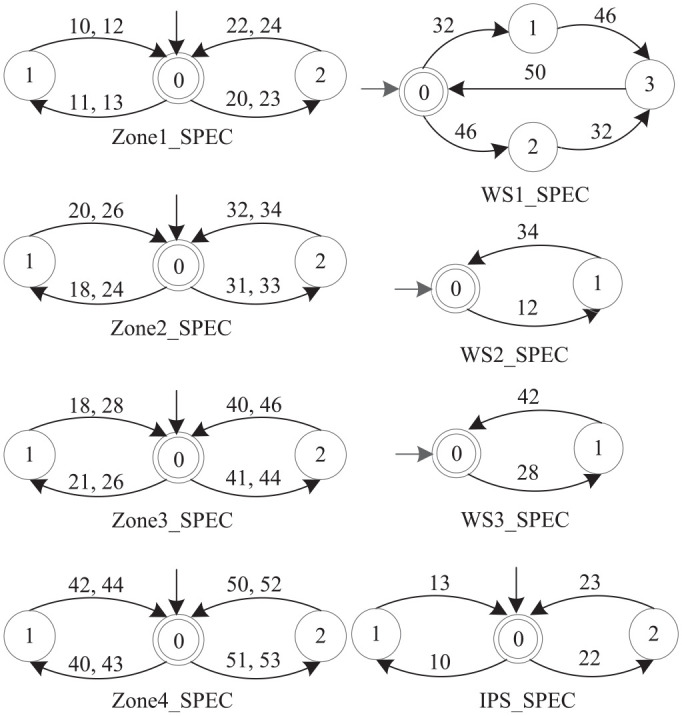
Eight specification automaton models for the AGVs system.

Compute the specification model **ZWISPEC** for the whole system by the synchronous product of all individual spec models in Figure 6.

On this basis, the supremal nonblocking supevisor of the AGVs system is

ZWISUP:=Supcon(AGV,ZWISUP)(4406,11338).

Namely, the full AGVs system consists of 4406 states and 11338 transitions.

The automaton model of the supervisor is too complex to be comprehended. We apply the proposed model abstraction method to simplify the supervisor model so that the control logic becomes clearer. Deadlock arises in many manufacturing systems when the number of being-processed parts in the system is more than a limit. We investigate this limit through the model abstraction method. Type 1 parts enter the system via event {11} and type 2 parts via event {21} . The completed products exit the system via event {51} . So we choose the observable event set as {11,21,51} , then compute the supremal quasi-congruence of the supervisor model ZWISUP using the method proposed in the paper. Finally, we obtain a simplified supervisor model for AGVs system with 22 cosets and 45 transitions.

**Figure 7.**
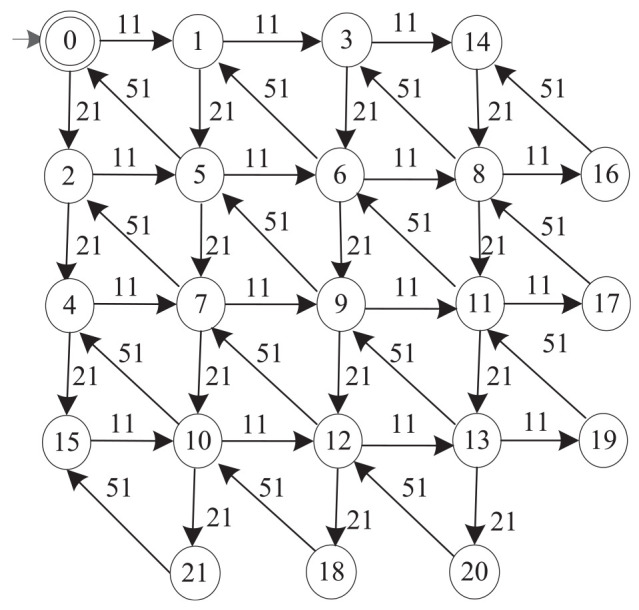
The quotient automaton of ZWISUP with respect to {11,21,51} .

Figure 7 shows the quotient automaton of ZWISUP with respect to an observable event set {11,21,51} . The simplified model reveals a constraint between events 11 and 21. Event 11 (21) can successively occur at most three times before an occurrence of event 12 (11). The reason is that after three successive occurrences of event 11 (12), there are three type 1 (2) parts in the manufacturing system. One is in WS1 waiting for the assembly with the other type of part, one is in AGV3 (AGV4) waiting to be transferred to WS1, and the last one is in WS2 (WS3). If event 11 (12) would occur once more before the other event occurs, AGV1 (AGV2) would have to park within the shared zone IPS, because WS2 (WS3) is full. Then AGV2 (AGV1) cannot enter the input station IPS2 (IPS1). The final product can never be assembled at WS1 and deadlock thus arises.

## Conclusions

In this paper, an optimization process is designed to automatically convert the computation of the coarsest quasi-congruence into a binary linear problem. Partitioning states to cosets is formalized as a binary allocation matrix $X_{n\times m}$ , where n is the number of states, m is an estimated number of cosets. Evidently m≤n . The task of finding the coarsest quasi-congruence is equivalent to the objective of finding the least number of cosets. To reduce the computation time, the translation process is also optimized by introducing fewer decision variables and simplifying constraints in the formulas. The computational efficiency and correctness of this method are verified by two different solvers. Therefore, the proposed method is not only a new solution for the coarsest quasi-congruence computation, but also provides us a more intuitive understanding of the quasi-congruence relation in the supervisory control theory.

A future direction is to apply more computationally efficient solvers, such as satisfiability modulo theories (SMT) and SAT, to compute the optimal state-to-coset allocation problem. Another direction is to apply the same method to compute model abstraction of finite automata with variables, namely the extended finite automata which are easily applicable to the real industrial scale problems.

## References

[1] Wonham WM Cai K . Supervisory control of discrete event systems. Cham: Springer, 2018.

[2] Cassandras CG Lafortune S . Introduction to discrete event systems. 2nd ed. Boston, MA: Springer, 2008.

[3] Ramadge PJ Wonham WM . Supervisory control of a class of discrete-event processes. SIAM J Control Optim 1987; 25(1): 206–230.

[4] Wonham WM Cai K Rudie K . Supervisory control of discrete-event systems: a brief history. Annu Rev Control 2018; 45: 250–256.

[5] Giua A Silva M . Petri nets and automatic control: a historical perspective. Annu Rev Control 2018; 45: 223–239.

[6] Lin F Wonham WM . On observability of discrete-event systems. Inf Sci 1988; 44(3): 173–198.

[7] Lin F Wonham WM . Decentralized control and coordination of discrete-event systems with partial observation. IEEE Trans Autom Control 1990; 35(12): 1330–1337.

[8] Schmidt K Moor T Perk S . Nonblocking hierarchical control of decentralized discrete event systems. IEEE Trans Autom Control 2008; 53(10): 2252–2265.

[9] Petri CA . Communication with automata. PhD Thesis, Air Force Systems Command Griffiss Air Force Base, New York, NY, 1996.

[10] Lefebvre D . Diagnosis with Petri nets according to partial events and states observation. IFAC Proc Vol 2012; 45(20): 1244–1249.

[11] Hu Q Du YY Yu SX . Service net algebra based on logic Petri nets. Inf Sci 2014; 268: 271–289.

[12] Sun DJ Chen YF Chen M , et al. On algebraic identification of critical states for deadlock control in automated manufacturing systems modeled with Petri nets. IEEE Access 2019; 7: 121332–121349.

[13] Li L Basile F Li ZW . An approach to improve permissiveness of supervisors for GMECs in time Petri net systems. IEEE Trans Autom Control 2020; 65(1): 237–251.

[14] Yang L Li ZW Giua A . Containment of rumor spread in complex social networks. Inf Sci 2020; 506: 113–130.

[15] Saboori A Hadjicostis CN . Verification of initial-state opacity in security applications of discrete event systems. Inf Sci 2013; 246: 115–132.

[16] Schmidt KW Breindl C . A framework for state attraction of discrete event systems under partial observation. Inf Sci 2014; 281: 265–280.

[17] Liu YY Cai K Li ZW . On scalable supervisory control of multi-agent discrete-event systems. Automatica 2019; 108.

[18] Chen QR Li Y Wu NQ , et al. Diagnosability of vector discrete-event systems using predicates. IEEE Access 2019; 7: 147143–147155.

[19] Wang DG Wang X Li ZW . State-based fault diagnosis of discrete-event systems with partially observable outputs. Inf Sci 2020; 529: 87–100.

[20] Zan X Wu ZP Guo C , et al. A pareto-based genetic algorithm for multi-objective scheduling of automated manufacturing systems. AIME Paper 2020; 12(1): 1–15.

[21] Li XB Nasr ESA EI-Tamimi AM , et al. Adaptive consensus of two coupled heterogeneous networked systems with bidirectional actions. IEEE Access 2020; 8: 35832–35841.

[22] Ma L Xu N Zhao X , et al. Small-gain technique-based adaptive neural output-feedback fault-tolerant control of switched nonlinear systems with unmodeled dynamics. IEEE Trans Syst Man Cybern Syst. Epub ahead of print 6 February 2020. DOI: 10.1109/tsmc.2020.2964822.

[23] Atampore F Dingel J Rudie K . Automated service composition via supervisory control theory. In: IEEE 13th International Workshop on Discrete Event Systems (WODES), Xi’an, China, 30 May–1 June 2016, pp.28–35. New York, NY: IEEE.

[24] Peeva K . Equivalence, reduction and minimization of finite automata over semirings. IEEE Trans Theor Comput Sci 1991; 88(2): 269–285.

[25] Billington J Gallasch GE Kristensen LM , et al. Exploiting equivalence reduction and the sweep-line method for detecting terminal states. IEEE Trans Syst Man Cybern Syst 2004; 34(1): 23–37.

[26] Flordal H Malik R Fabian M , et al. Compositional synthesis of maximally permissive supervisors using supervisor equivalence. Discrete Event Dyn Syst 2007; 17(4): 475–504.

[27] Su R van Schuppen JH Rooda JE . Model abstraction of nondeterministic finite-state automata in supervisor synthesis. IEEE Trans Autom Control 2010; 55(11): 2527–2541.

[28] Ma C Wonham WM . Nonblocking supervisory control of state tree structures. IEEE Trans Autom Control 2006; 51(5): 782–793.

[29] Wang DG Wang X Li ZW . Nonblocking supervisory control of state-tree structures with conditional-preemption matrices. IEEE Trans Industr Inform 2020; 16(6): 3744–3756.

[30] Peled D . Partial order reduction: model-checking using representatives. In: IEEE symposium on mathematical foundations of computer science (eds Penczek W Szałas A ), Crakow, Poland, 2–6 September 1996, pp.93–112. Berlin: Springer.

[31] Wong KC Wonham WM . On the computation of observers in discrete-event systems. Discrete Event Dyn Syst 2004; 14(1): 55–107.

[32] Feng L Wonham WM . On the computation of natural observers in discrete-event systems. Discrete Event Dyn Syst 2010; 20(1): 63–102.

[33] Benson DB Shachar OB . Bisimulation of automata. Inform Comput 1988; 79(1): 60–83.

[34] Paige R Tarjan RE . Three partition refinement algorithms. SIAM J Comput 1987; 16: 973–989.

[35] Hashimoto J . On meromorphisms and congruence relations. J Math Soc Jpn 1967; 19(1): 70–81.

[36] Wong KC Wonham WM . Hierarchical control of discrete event systems. Discrete Event Dyn Syst 1996; 6(3): 241–273.

[37] Wong KC . On the complexity of projections of discrete-event systems. Discrete Event Dyn Syst 1998; 1: 201–208.

[38] Fernandez JC . An implementation of an efficient algorithm for bisimulation equivalence. Sci Comput Program 1990; 13: 219–236.

[39] Zhang RY Gan YM Chao WJ , et al. Improved algorithm of quasi-congruence in discrete-event system. IET Control Theory Appl 2012; 29: 151–156.

[40] Kordafahar MS Jaberi N Rafeh R . On the optimization of CPLEX models. Comput Sci 2013; 4(9): 2810–2816.

[41] Zhou NF Kjellerstrand H . Optimizing SAT encodings for arithmetic constraints. In: IEEE conference on principles and practice of constraint programming, Melbourne, VIC, Australia, 28 August–1 September 2017, pp.671–686. New York, NY: IEEE.

[42] Boyer V Didier ED . Recent advances on GPU computing in operations research. In: IEEE 27th symposium on parallel & distributed processing workshops & PhD forum, Boston, MA, 20–24 May 2013, pp.1778–1787. New York, NY: IEEE.

[43] Ralphs T Shinano Y Berthold T , et al. Parallel solvers for mixed integer linear optimization, chapter 8. Cham: Springer International Publishing, 2018. pp.283–336.

[44] Ciavotta M Ardagna D Gibilisco GP . A mixed integer linear programming optimization approach for multi-cloud capacity allocation. J Syst Softw 2017; 123(1): 64–78.

[45] Feng L Wonham WM . TCT: a computation tool for supervisory control synthesis. In: Proceedings of the 8th international workshop on discrete event systems – WODES, Ann Arbor, MI, 10–12 July 2006, pp.388–389.

[46] Ramadge PJG Wonham WM . The control of discrete event systems. Proc IEEE 1989; 77(1): 81–98.

[47] Rabin MO Scott D . Finite automata and their decision problems. IBM J Res Dev 1959; 3(2): 114–125.

[48] Hopcroft JE Motwani R . Introduction to automata theory, languages, and computation. 2nd ed. Reading, MA: Addison Wesley, 2012.

[49] Maclane S Birkhoff G . Algebra. Providence, RI: AMS Chelsea Publishing, 1988.

[50] Feng L . Computationally efficient supervisor design in discrete-event systems. PhD Thesis, University of Toronto, Toronto, ON, Canada, 2008.

[51] Feng L Wonham WM . Supervisory control architecture for discrete-event systems. IEEE Trans Autom Control 2008; 53(6): 1449–1461.

[52] Jeroslow RG Wang JC . Solving propositional satisfiability problems. Ann Math Artif Intell 1990; 1(1–4): 167–187.

[53] Weber T. A SAT-based Sudoku solver. In: IEEE LPAR-12 conference on logic for programming, artificial intelligence, and reasoning, Maun, Botswana, 7–12 May 2017, pp.11–15.

[54] Zhang X Feng L Törngren M , et al. Formulating customized specifications for resource allocation problem of distributed embedded systems. In: Proceedings of the 35th International Conference on Computer-Aided Design (ICCAD), Austin, TX, 7–10 November 2016, pp.1–8.

[55] Kuendee P Janjarassuk U . A comparative study of mixed-integer linear programming and genetic algorithms for solving binary problems. In: 5th International Conference on Industrial Engineering and Applications (ICIEA), Singapore, 26–28 April 2018, pp.284–288.

